# Protruding Features of Viral Capsids Are Clustered on Icosahedral Great Circles

**DOI:** 10.1371/journal.pone.0152319

**Published:** 2016-04-05

**Authors:** David P. Wilson

**Affiliations:** Department of Physics, Albion College, 611 E. Porter St., Albion, Michigan, United States of America; Academia Sinica, TAIWAN

## Abstract

Spherical viruses are remarkably well characterized by the Triangulation (T) number developed by Casper and Klug. The T-number specifies how many viral capsid proteins are required to cover the virus, as well as how they are further subdivided into pentamer and hexamer subunits. The T-number however does not constrain the orientations of these proteins within the subunits or dictate where the proteins should place their protruding features. These protrusions often take the form of loops, spires and helices, and are significant because they aid in stability of the capsid as well as recognition by the host organism. Until now there has be no overall understanding of the placement of protrusions for spherical viruses, other than they have icosahedral symmetry. We constructed a set of gauge points based upon the work affine extensions of Keef and Twarock, which have fixed relative angular locations with which to measure the locations of these features. This work adds a new element to our understanding of the geometric arrangement of spherical viral capsid proteins; chiefly that the locations of protruding features are not found stochastically distributed in an icosahedral manner across the viral surface, but instead these features are found only in specific locations along the 15 icosahedral great circles. We have found that this result holds true as the T number and viral capsids size increases, suggesting an underlying geometric constraint on their locations. This is in spite of the fact that the constraints on the pentamers and hexamer orientations change as a function of T-number, as you need to accommodate more hexamers in the same solid angle between pentamers. The existence of this angular constraint of viral capsids suggests that there is a fitness or energetic benefit to the virus placing its protrusions in this manner. This discovery may have profound impacts on identifying and eliminating viral pathogens, understanding evolutionary constraints as well as bioengineering for capsid drug delivery systems. This result also suggests that in addition to biochemical attachment restrictions, there are additional geometric constraints that should be adhered to when modifying protein capsids.

## Introduction

**Fig 1:**
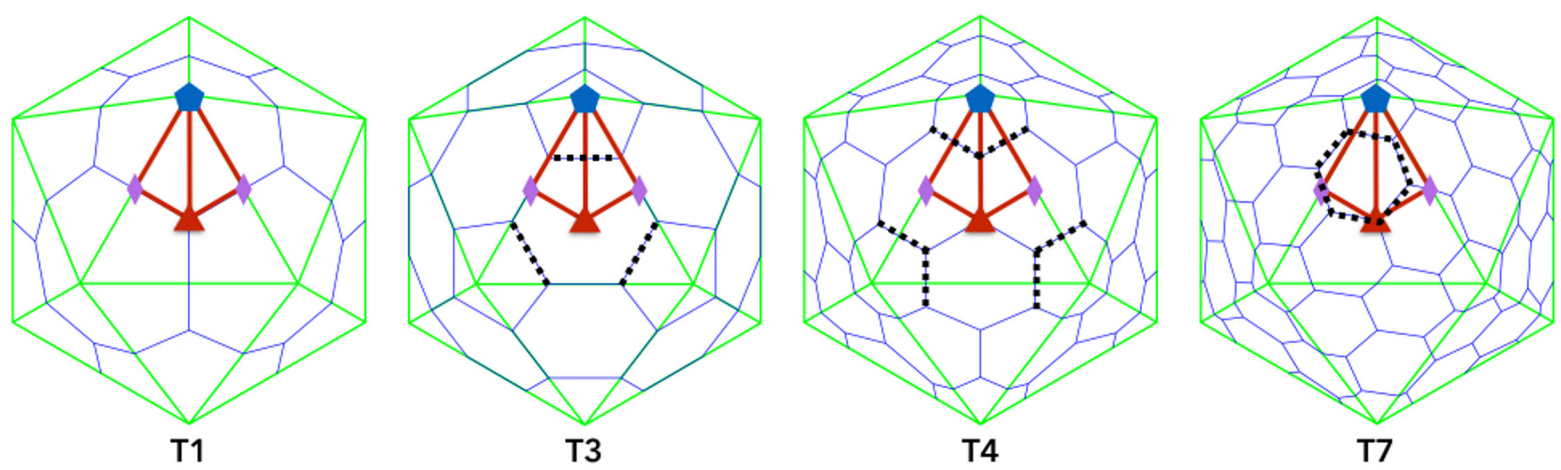
Triangulation numbers and Constraints. The standard Triangulation number architecture for spherical capsids ranging from T1 to T7 capsids. Each are composed of 12 pentamers (5-protein units) and a variable number of hexamers (6-protein units). The Asymmetric Unit (AU) for each capsid is shown as a red kite stretching from the 5-fold (blue pentagon) to each of the 2-folds (red diamonds) to the 3-fold axes (green triangle). The icosahedral great circles also encompass each of the 5 distinct red lines shown. As the triangulation number increases, the hexamers must rearrange as seen, which changes the restrictions on their protrusions, shown as dashed lines above. While the capsid proteins are considered to be quasi-equivalent, they are not for the purpose of protrusions. The dotted lines show where modifications for bioengineering will likely be less favorable, except where they intersect with the boundaries of the AU (*i.e*. the great circles). In general it also appears the p23 within the AU is less favorable. The icosahedral capsids were drawn using the Icosahedral Server [3].

Viruses are classified as helical, spherical or irregular, depending on the shape of their viral capsid which encapsulates and protects the genetic material of the virus, as well as aids with infection of the host. In this paper we focus on spherical viruses which are known to have icosahedral symmetry. These capsids have also been remarkably well classified by their Triangulation (T) number developed by Casper and Klug [1] which states that all capsid proteins have nearly identical chemical environments, known as quasi-equivalence. The triangulation number specifies that there are T-proteins within the asymmetric unit and that (60*T*) copies of these proteins are required to complete the spherical capsid. In addition, the T-number specifies how the capsid proteins should be further subdivided into the two major subunits, the pentamers and hexamers. There are always at least 12 pentamer and 10(*T* − 1) hexamer subunits. The pentamers are arranged around the 5-fold symmetry axes and the hexamers are packed into the area between the pentamers, see Fig 1. In general as the T-number increases, so does the radius of the virus, however because the angle between adjacent 5-fold axes is the same for all icosahedral capsids at 63.4°, the angle between the pentamers must remain fixed as well [1, 2], leading to constraints on the hexamer packing.

Most spherical capsids have prominent protruding features which take the form of loops, spires, helices and/or bulges. These protrusions, defined to be the most radially distal structures, can be composed of single or multiple proteins and can serve as potential targets for bioengineering surface protein modifications. Many protrusions are known to have biological functions, such as the immuno-dominant regions which lie at the top of the alpha-helical bundles on the surface of HepB [4, 5] as well as the attachment mediating protrusions of adeno-associated virus [6].

Additionally these features often play a role in stability of the capsid, as well as recognition by the host [7, 8] and have been suggested to play an important role in capsid stabilization [9]. Some examples of protruding features include convex protein-protein boundaries (e.g. L-A, CCMV), single protein bulges (CCMV), single protein loops (MS2, GA), multi protein helical bundles (HepB), multi-protein spires (PAV), pentamers (SV40) and as portions of the hexamers (HK97 Prohead II).

Until now the the specific locations of spherical capsid protrusions has been poorly understood as their locations area not specified by T-number nor icosahedral symmetry. Additionally, the triangulation number does not impose any spatial or angular constraints on the locations of the protruding features, nor on the specific arrangement of the proteins within the pentamers and hexamers. Understanding the locations of these features as well as the constraints on their placement could have a profound impact on our understandings of virus evolution and pathogenicity, as well as our ability to modify viral capsids for drug delivery systems.

In order to understand the spatial distributions of these protruding features, we measured their locations relative to a set of fixed gauge points, which have a fixed angular location on the sphere making them a natural coordinate system. These gauge points were derived from the set of all possible affine extensions of the icosahedral point sets derived by Keef and Twarock [10]. The gauge points lie upon the 15 icosahedral great circles, see Table 1, making them a natural tool for measuring and characterizing spherical capsids as they can be used to track changes throughout maturation as well as to quantify individual structural feature differences between capsids. Recent work by Twarock et al. [11, 12] and Janner [13] demonstrated that many spherical viruses conform to the affine extended icosahedral symmetry point arrays. The vertices of these point arrays represent material boundaries of the proteins, genetic material as well as their interfaces. Each point arrays imposes a different set of radial and angular constraints on the virus. The gauge points are built from the outmost points of these arrays, and are used to determine their only free parameter, radial scaling.

**Table 1:** Spherical Coordinate Locations of the Gauge Points.

Gauge Points			
GP	*θ*	*ϕ*	Plane
1	0.0°	31.7°	5-fold
2	8.3°	20.7°	P53
3	10.8°	16.9°	P53
4	13.3°	12.9°	P53
5	15.5°	9.4°	P53
6	20.9°	0.0°	3-fold
7	15.5°	0.0°	P32
8	14.5°	0.0°	P32
9	13.3°	0.0°	P32
10	12.2°	0.0°	P32
11	10.8°	0.0°	P32
12	9.7°	0.0°	P32
13	7.3°	0.0°	P32
14	5.2°	0.0°	P32
15	0.0°	0.0°	2-fold
16	0.0°	8.3°	P25
17	0.0°	11.6°	P25
18	0.0°	15.5°	P25
19	0.0°	17.2°	P25
20	0.0°	19.3°	P25
21	0.0°	20.9°	P25

**Fig 2:**
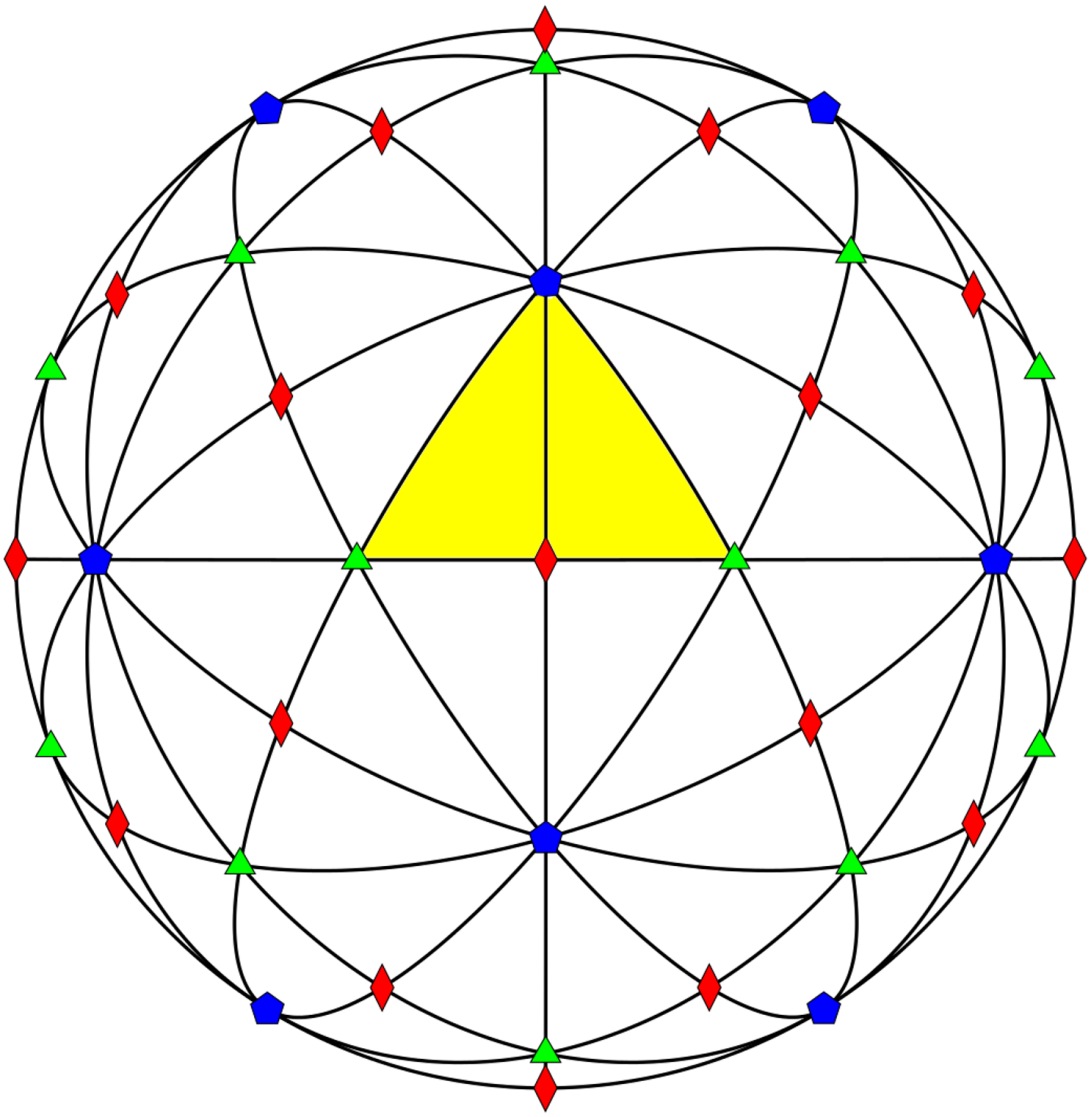
Icosahedral Great Circles. A view down the 2-fold axes of the 15 icosahedral great circles encompassing the 2-fold (red diamond), 3-fold (green triangle), 5-fold (blue pentagon) symmetry axes, respectively. We found that protruding features cluster on these circles, and are seldom found in the white regions between them, see Fig 3. The spherical area can be subdivided into 60 identical units, known as the Asymmetric Unit (AU), shown here as a yellow (shaded) triangle. Each circle passes through the (5-3-2-3-5-2-5-3-2-3-5-2-5*) fold axes, where 5* indicates the full cycle. The number of intersecting circles determines the symmetry of the point, 2-folds are diamonds, 3-folds are triangles, and 5-folds are pentagons. Additionally that all the gauge points lie on the border of the AU and plane bisecting it and thus are found on the great circles, see Table 1. Note that the full icosahedral group, which includes mirror symmetry, would only required the right hand side of the kite, and would have 120 fundamental domains, also known as the Coxeter Group *H*3.

The angular locations of the 21 gauge points within the asymmetric unit are given in spherical coordinates, see Figs 4 and 5. Our polar angle is *ϕ* and our azimuthal angle is *θ*. The +*z* axis is aligned with a 2-fold axis, seen here as gauge point 15, and our +*x* axis is in the plane containing the +*z* and 5-fold of the asymmetric unit, which contains gauge points 1 and 15-21, see Figs 2 and 4. All gauge points lie on icosahedral great circles. Each gauge point is also associated with two or more distinct affine extended point arrays.

**Fig 3:**
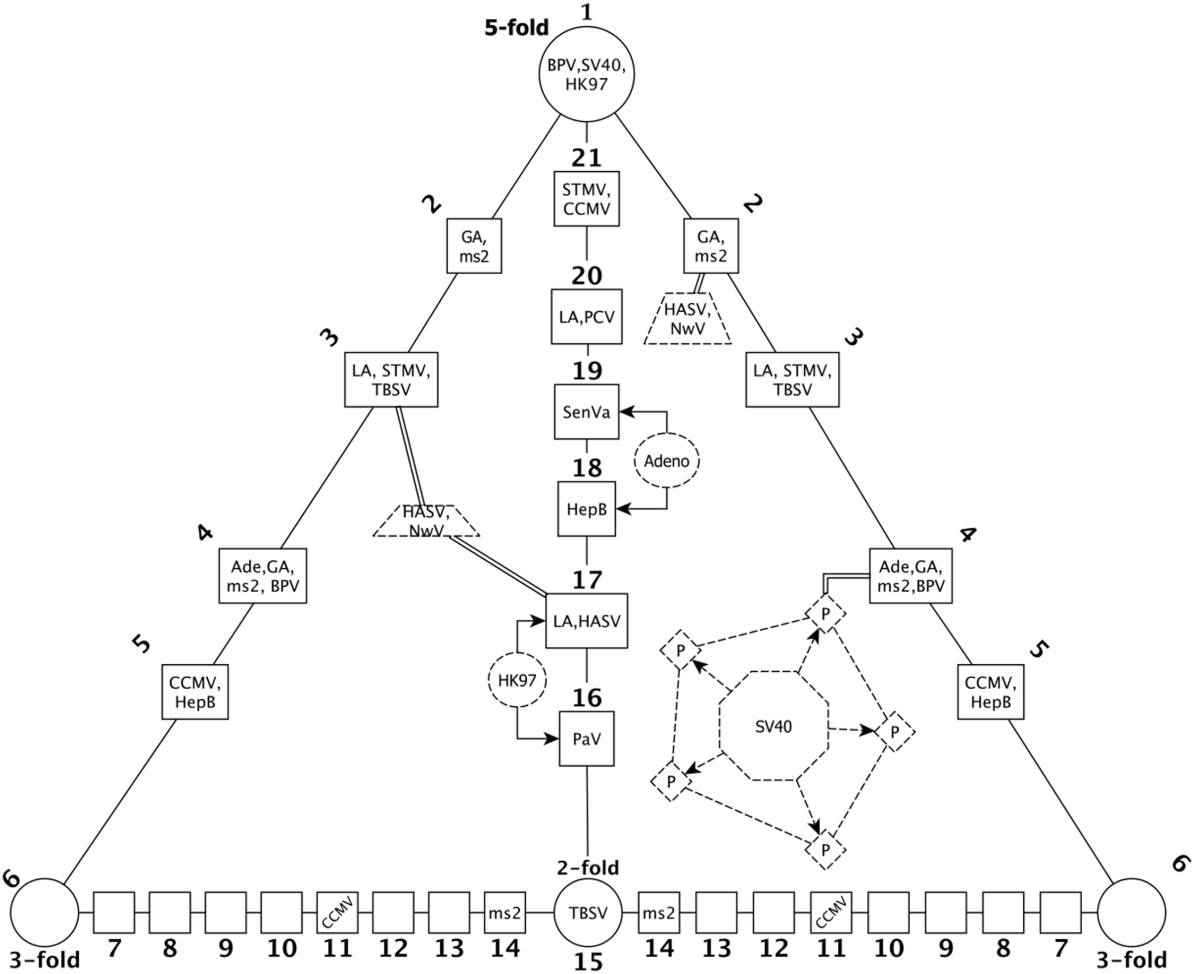
Gauge Points. A schematic of the 21 gauge points within the asymmetric unit which lie between the 2, 3 or 5 fold axes. Gauge points 2 – 14 appear twice as they border the AU, however points 2-6 are equivalent up to a rotation about the 5-fold axes and points 6-14 are equivalent up to a rotation about the 2-fold axes. The angular locations of the gauge points are given in Table 1. The associated gauge points of each virus are indicated as per Table 2. We did not found any viruses with protruding features near gauge points 6-10, 12 and 13.

In this paper we will show that nearly all protruding features of spherical viruses are found on or near the icosahedral great circles which subtend the icosahedral symmetry axes, leaving the majority of the viral capsid unused for protrusions. This result is intriguing, as it is not required by icosahedral symmetry, nor quasi-equivalence; as in principle different viruses could distribute their protrusions stochastically around the sphere while maintaining icosahedral symmetry, see Figs 2 and 3. Moreover even the angular locations of the protrusions are not continuously distributed, but instead are clustered near the gauge points of the affine extended icosahedral symmetry point arrays derived by Keef and Twarock [10]. The determination of the appropriate gauge points is important to understanding the affine extended symmetry of viruses, as these points dictate the allowable internal point array structures [10].

## Methods

**Fig 4:**
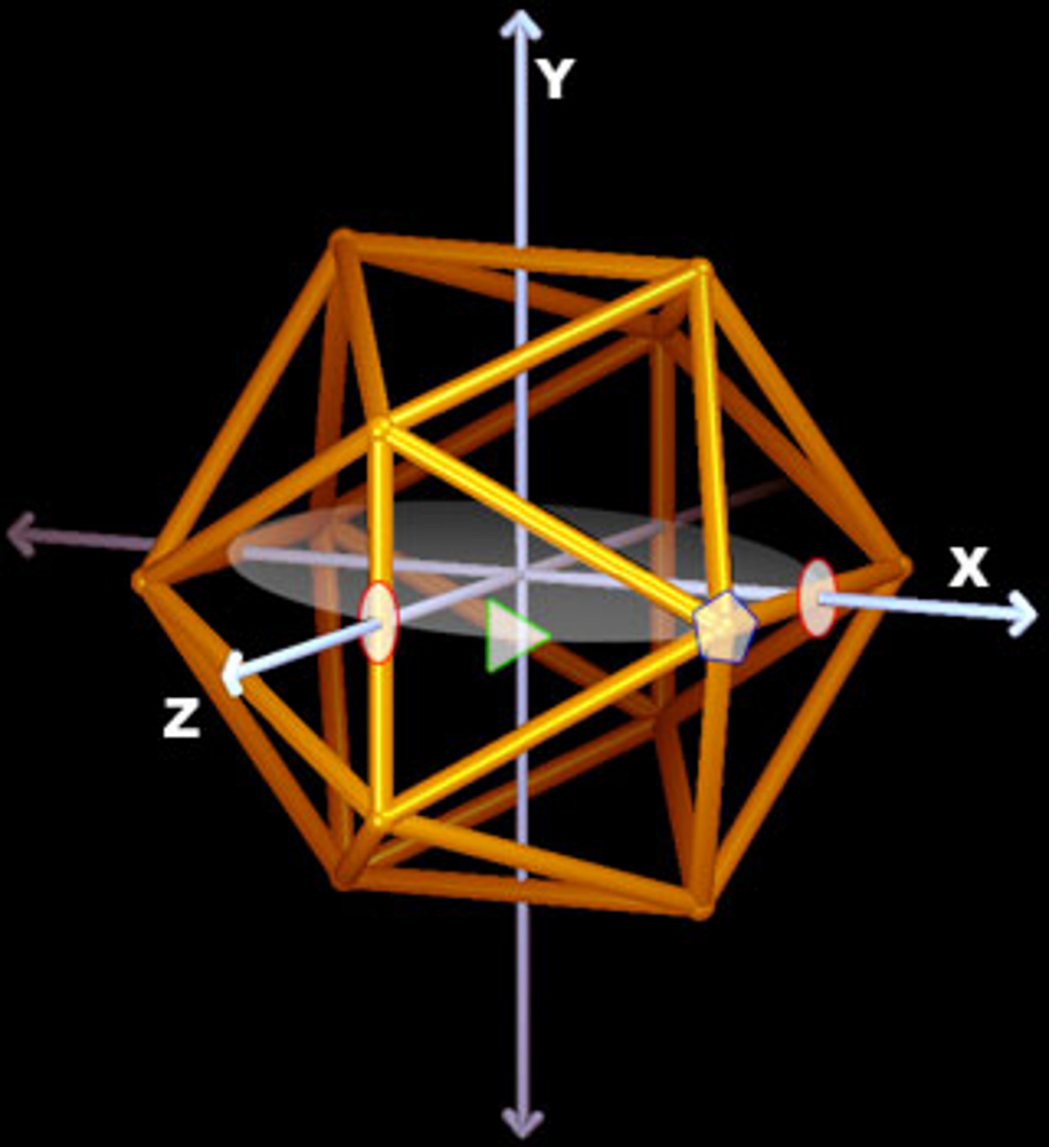
Asymmetric Unit. The standard orientation of the icosahedral capsid in the Viper Database [3]. The 2-fold axes are shown as red ovals, the 3-fold axes as green triangles and the 5-folds as blue pentagons. We will use this orientation to measure out spherical angles in Table 1 with *ϕ* being measured from +*z* axis towards the *x* − *y* plane and *θ* from the +*z* axis in the *x* − *z* plane. While the asymmetric unit (AU) is not uniquely defined it always contain the volume created by the intersection of three planes containing adjacent icosahedral symmetry vectors, referred to as p52, p53 and p32.

We composed our virus capsid data set of a wide range of viruses from the literature, spanning T = 1 to T = 7 capsids and we also included several quasi-T number viruses. Icosahedral capsids may be divided into 60 equivalent, irreducible sections, known as the asymmetric unit (AU), which when you apply the icosahedral rotations will reconstruct the entire viral capsid, similar to a unit cell in crystallography, see Figs 1 and 4. Our analysis begins by finding the protruding features for each viral capsid that are contained in or neighboring the asymmetric unit and then we compute their angular separation from the set of gauge points, see Table 2.

**Table 2:** Relative Angular Locations of Protruding Features to their Associated Gauge Points.

Protruding Features			
Virus	T	Gauge Point(s)	Angle
Adenovirus [21]	1	4	4.2°
		18 & 19	2.6°
LA Virus [22]	1	3	2.1°
		17	1.8°
		20	0.5°
PCV [23]	1	20	1.5°
STMV [24]	1	3	2.1°
		21	2.6°
CCMV [16]	3	5	3.2°
		11	3.3°
		21	4.3°
CCMV Swollen [25]	3	5	1.4°
		11	1.4°
		21	5.4°
GA [26]	3	2	1.9°
		4	2.2°
MS2 [15]	3	2	2.2°
		4	2.6°
		14	2.8°
PAV [27]	3	16	0.5°
TBSV [28]	3	3	1.7°
		15	2.3°
Seneca Valley [29]	pT3	19	2.0°
HASV [30]	4	2	3.7°
		17	6.2°
HepB [31]	4	5	0.5°
		18	0.4°
HepB ADYW [32]	4	5	0.6°
		18	0.3°
N*ω*V [33]	4	2	3.7°
		3	5.9°
SV40 [34]	7d	1	3.5°
		4	6.7°
BPV-Type 1 [35]	7d	1	0.0°
		4	5.6°
HK97 Prohead II [36]	7l	1	0.0°
		16 & 17	3.1°
HK97 Head II [37]	7l	1	0.05°

We see clustering of the external features of many spherical viruses with the planes which contain the fold symmetries of the icosahedral group. For a sense of scale, the angle between the 5-fold and 2-fold is 31.7°, the angle between the 2-fold and 3-fold is 20.9° and the angle between $\vec{5}$ and $\vec{3}$ is 37.4°.

### Gauge Points

**Fig 5:**
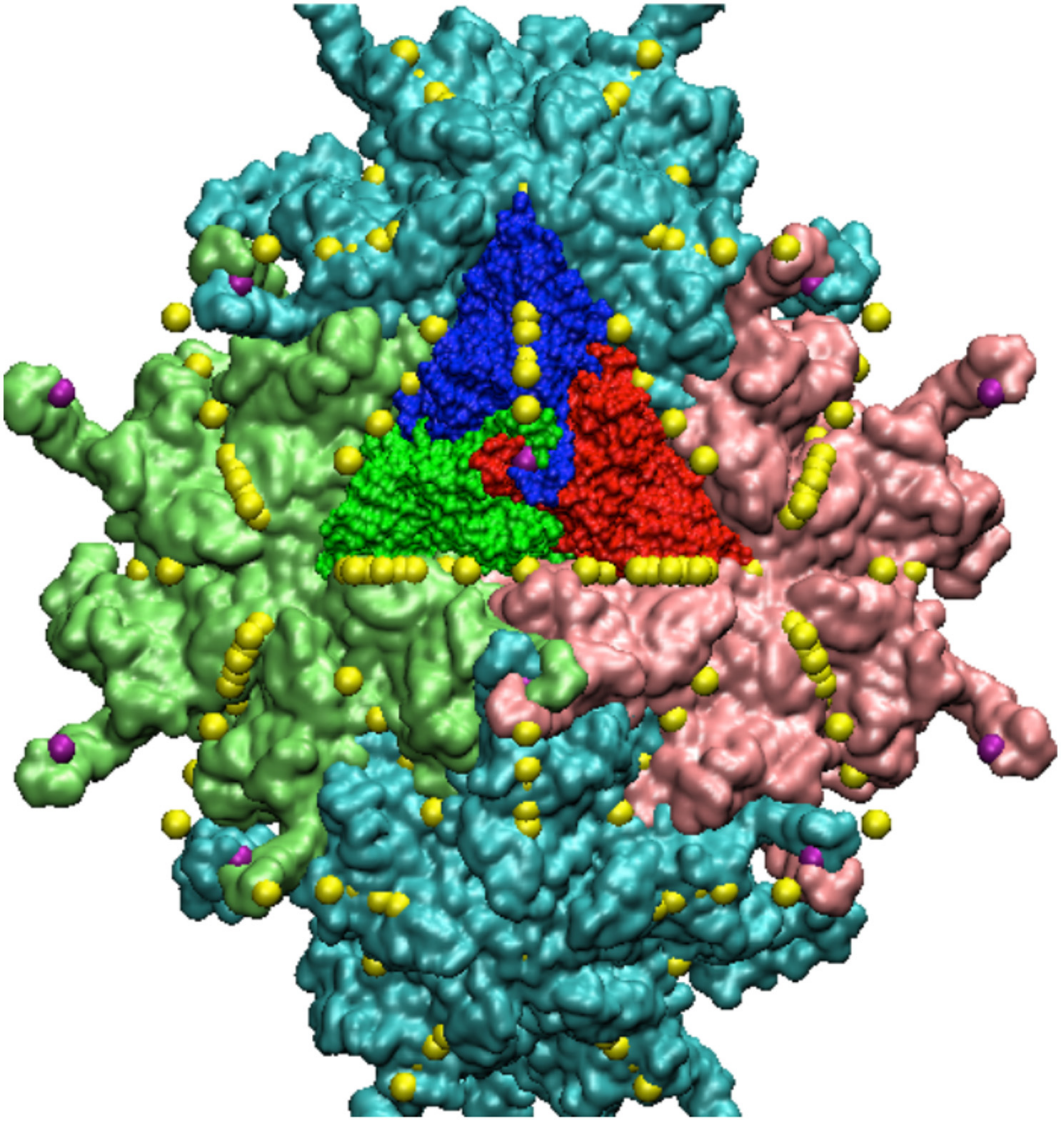
Section of PAV. A section of the full PAV capsid showing two pentamers (light blue) and two hexameters (pink and green). All 21 gauge points of the AU are displayed as yellow spheres, the 16th gauge point is shown as purple. The 16th gauge point is also known as the local 3-fold, in reference to the AU.

We construct our set of gauge points by including all possible outer hulls of the affine extensions of the icosahedral point sets derived by Keef and Twarock [10]. Careful examination of the gauge points reveal that they all lie upon the 15 icosahedral great circles; which are defined as the subset of great circles on the sphere which transverse any two adjacent icosahedral symmetry axes (2, 3 or 5-fold), see Fig 2. There are 21 unique gauge points within the AU, see Fig 5, and their coordinates are given in Table 1.

We determine the radially scaling of the gauge points by initially scaling all points such that they are 5Å beyond the outermost atom of the viral capsid. We then lower each gauge points until they intersect or pass through the viral capsid surface, which is approximated by allowing each atom to have a van der Waals radius of 2Å. Gauge points which do not come into contact with the viral surface, due to gaps and points that are not at 95% or above, are not considered admissible points and are disregarded.

### Protruding Features

We find the protruding features by selecting the outermost atoms (top 5% by radius) of the viral capsid, referred to as the peak atoms. We then group each peak atom with all other atoms within a given angular cutoff, which we refer to as a wedge. Next we compute the average surface radius of each wedge and look at the atoms above a cutoff, given by *R*_*cut*_ = .5(*R*_*surf*_ + *R*_*peak*_). We then repeat this algorithm for all of the peak atoms and if distinct features overlap, our algorithm separates them out, based on their proximity to the center of mass of each wedge. Finally, we look for any features near the 95% of the peak radii that are not yet included and created wedges around them as well. The angular cutoff of the wedge is chosen to be the largest angular separation between the set of gauge points, see Table 1. The algorithm is very robust, and we have yet to find a virus that is not properly characterized by it.

**Fig 6:**
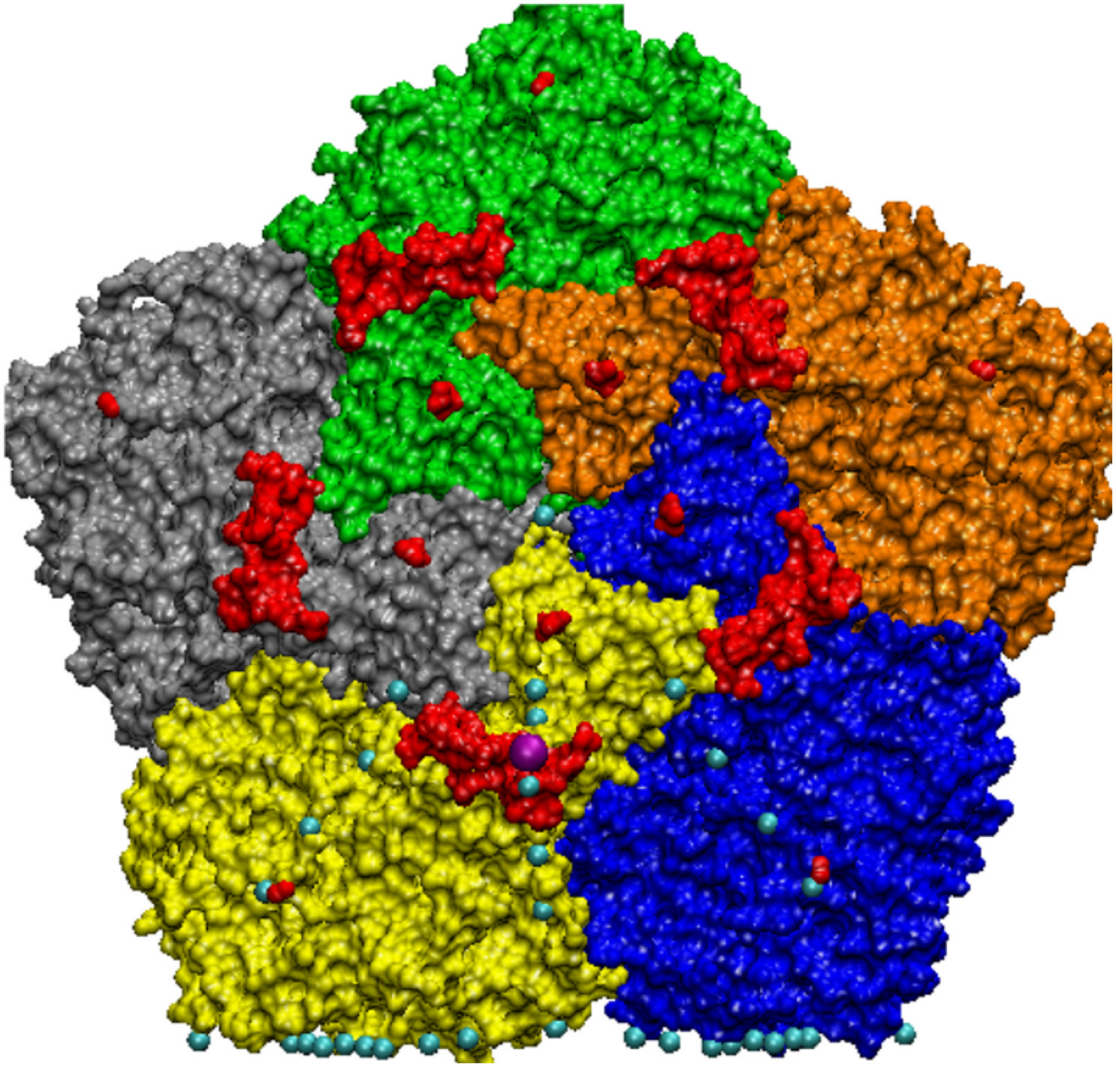
Seneca Valley—is a pseudo T3 virus, with all of its protrusions shown in red. Some of these protrusions are very minor, being composed of only a few atoms and their results are not reported here. All of the gauge points within the AU are shown in cyan and the gauge point identified with its major protrusion is shown in purple.

Once the protruding features have been found, we measure their location on the sphere by using our set of gauge points. We determine the nearest gauge points by computing the angle between the center of mass of the protrusion and the position vector of each gauge point. Finally we check the radial scaling of the nearest gauge points to make sure they are above the 95% radius level, which is true of all the viruses we’ve studied, see Table 2. Occasionally the algorithm finds minor protrusions, which are composed of only a few atoms, see Fig 6 and we did not report those features in our results. Previous measures of external features measured the linear distance from center of mass of the feature to the gauge points [11], which could vary by several angstroms based upon your choice of radial cutoff, whereas our angular measure is fairly insensitive to the cutoff, making it much more robust.

**Fig 7:**
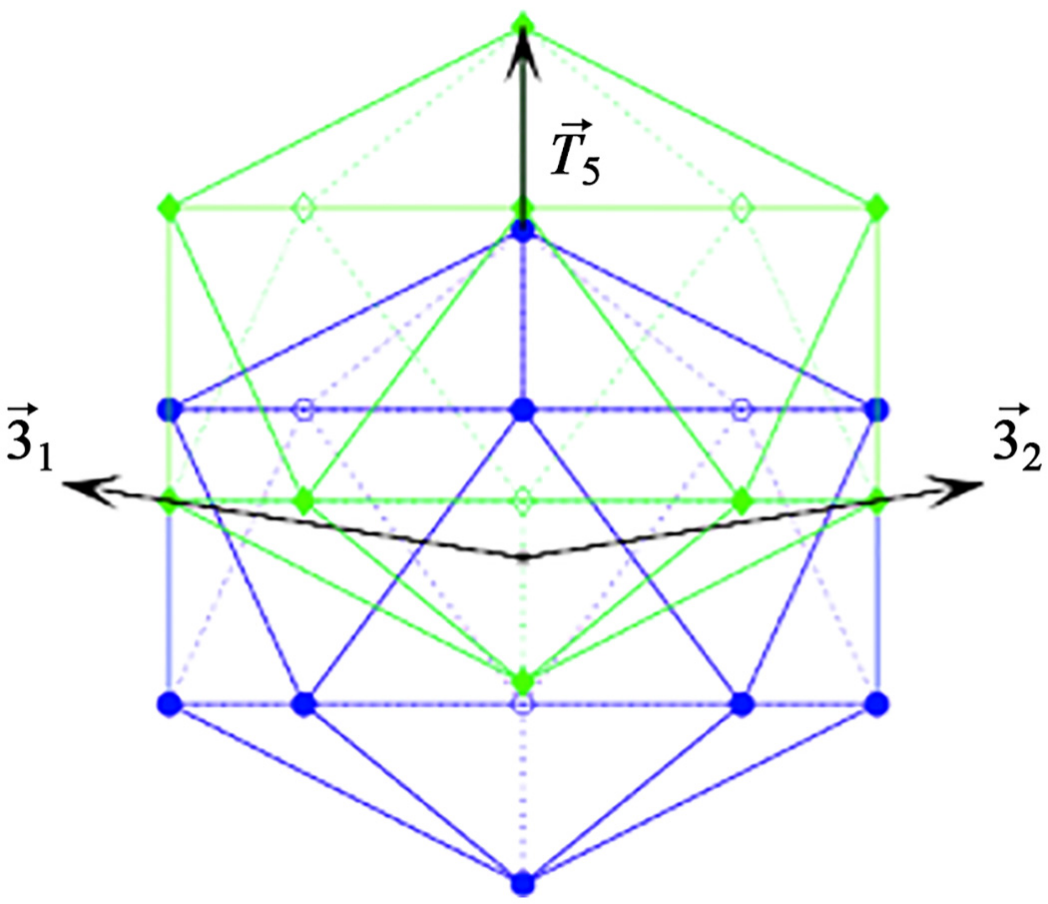
Affine Extensions. We construct the affine extensions of the base icosahedral vertex sets by first translating the polyhedra along one of its symmetry axes, and then applying icosahedral symmetry to the displaced structure. Here we show the base icosahedron (blue) being translated along the 5-fold axes $\left({\vec{T}}_{5}\right)$ to form a new icosahedron (green) displaced from the origin. Once icosahedral symmetry about the blue icosahedron is applied, the displaced vertices create new polyhedra at different radii. The displacement lengths are chosen such that at least two of the original vertices intersect the original symmetry axes or intersect neighboring displaced vertices [10]. In this way, we construct a new icosahedral vertex set, adding a radial component to the original icosahedral symmetry. In this example, we intersect the 3-fold axes $\left({\vec{3}}_{1,2}\right)$ and thus will create a new larger radius dodecahedron. The top vertex of the original icosahedron which now resides at the tip (green) of ${\vec{T}}_{5}$ will be the largest radius point, and thus the gauge point of this affine extension.

The affine extended point arrays are created by displacing the vertices of the the icosahedral polyhedra, namely the icosahedron, dodecahedron and icosadodecahedron along the icosahedral symmetry vectors by an length *α*. The vertices are displaced until at least two of the translated vertices intersect with symmetry axes, which recreates the icosahedral symmetry at a new radius, or they intersect neighboring displaced vertices. After translation, you then apply the icosahedral group rotations, forming the point array, see Fig 7. The effect of this translation is to extend a rotation symmetry at a single radius to a rotation symmetry at many radii in a mathematically consistent way which preserves the group structure at each radial level [10]. We construct our set of gauge points by considering the most radially distal points from all possible affine displacements of the icosahedral polyhedra.

## Results

We have identified 38 protruding features within the asymmetric unit (AU), from from 20 distinct spherical viruses ranging from T1 to T7 capsids. We have computed the angular separations between these features and their nearest gauge points, which appear in Tables 2 and 1, respectively. We have selected these viruses to be representative of a range of T-numbers from recent papers [11, 12], as well as a few suggested to the authors.

**Fig 8:**
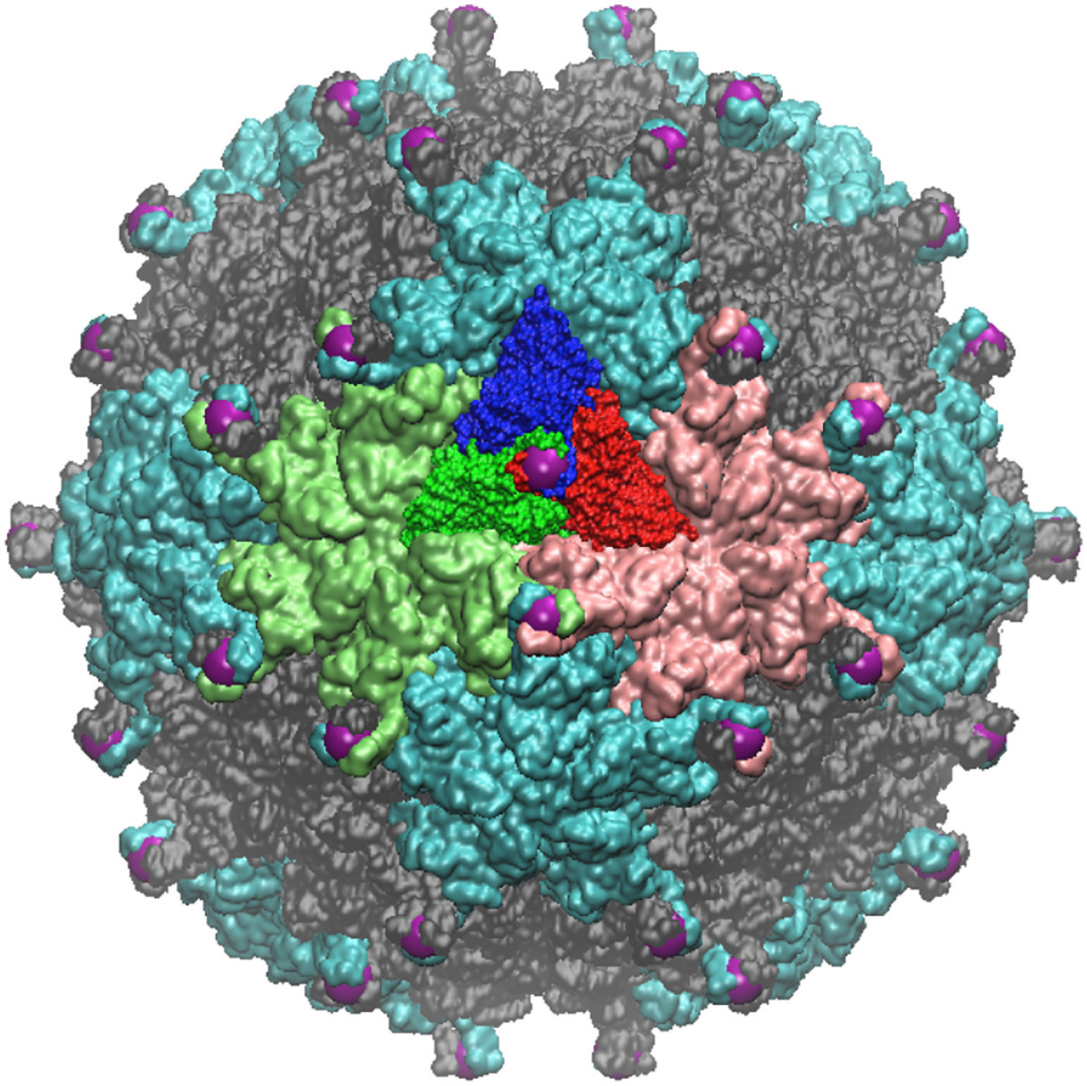
Pariacoto Virus. The full PAV (T3) Capsid is made up of 180 identical proteins. It is formed by applying 60 icosahedral rotations of the asymmetric unit (3 proteins in a triangular shape, blue, red and dark green). The blue protein when rotated about the 5-fold axis (gauge point 1) forms a pentamer (5 proteins), of which there are 12 shown in light blue. The red and dark green proteins form pink and green hexamers (6 proteins) when rotated about the 3-fold axes (gauge point 6). The pink and green hexameters meet at a 2-fold axes (gauge point 15). In total there are 20 hexamers, shown in gray. The 16th gauge point, which lies between the 5 and 2 fold axes best describes the outermost features of PAV and is shown as a purple sphere, see Fig 2. showing the alignment of the 16th gauge point (purple sphere) with the outermost features of PAV, which are towers of 3 twisted proteins.

Protruding features take on various forms *e.g.* as single protein structures (*e.g.* loops of MS2), coordinated multi-protein structures (*e.g.* helical bundles of HepB and three protein wrapped tower of PAV, see Fig 8) and as the boundary of two or more proteins. Many viruses have several distinct protrusions found within the asymmetric unit (AU) and each feature is reported in Table 1. Additionally, some protruding features are approximately equidistant from two gauge points, *e.g.* adeno and HK97, and therefore have two gauge points listed. The majority of protruding features were found to be within 3.5° of their respective gauge points, with several being nearly perfectly aligned, *e.g.* LA, PAV and HepB. These results are intriguing as the location of protruding features is not dictated by icosahedral symmetry nor the Triangulation number; and in principle could occur anywhere within the asymmetry unit.

**Fig 9:**
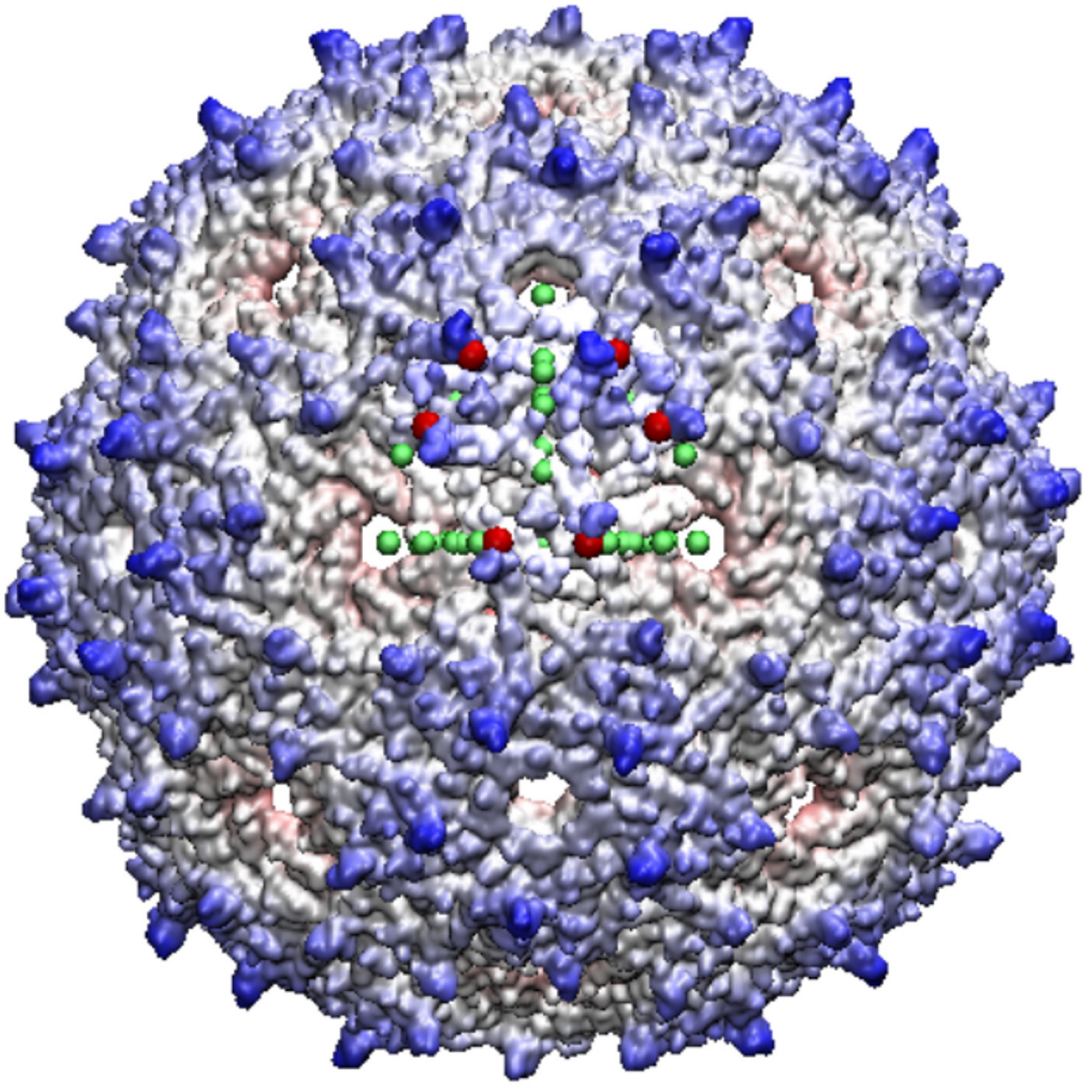
Bacteriophage MS2—is a T3 virus with three surface loops within the AU, however there are 3 additional loops adjacent to the AU on the 5-3 and 2-3 planes. The capsid is colored radially with the protrusions shown in blue, the nearest gauge points are red spheres with the other gauge points of the AU are shown for reference as green. GA virus is very similar to MS2, and the bottom two loops of GA are lying down near the capsid surface and is not considered as a protrusion, a feature easily noticed using gauge points.

**Fig 10:**
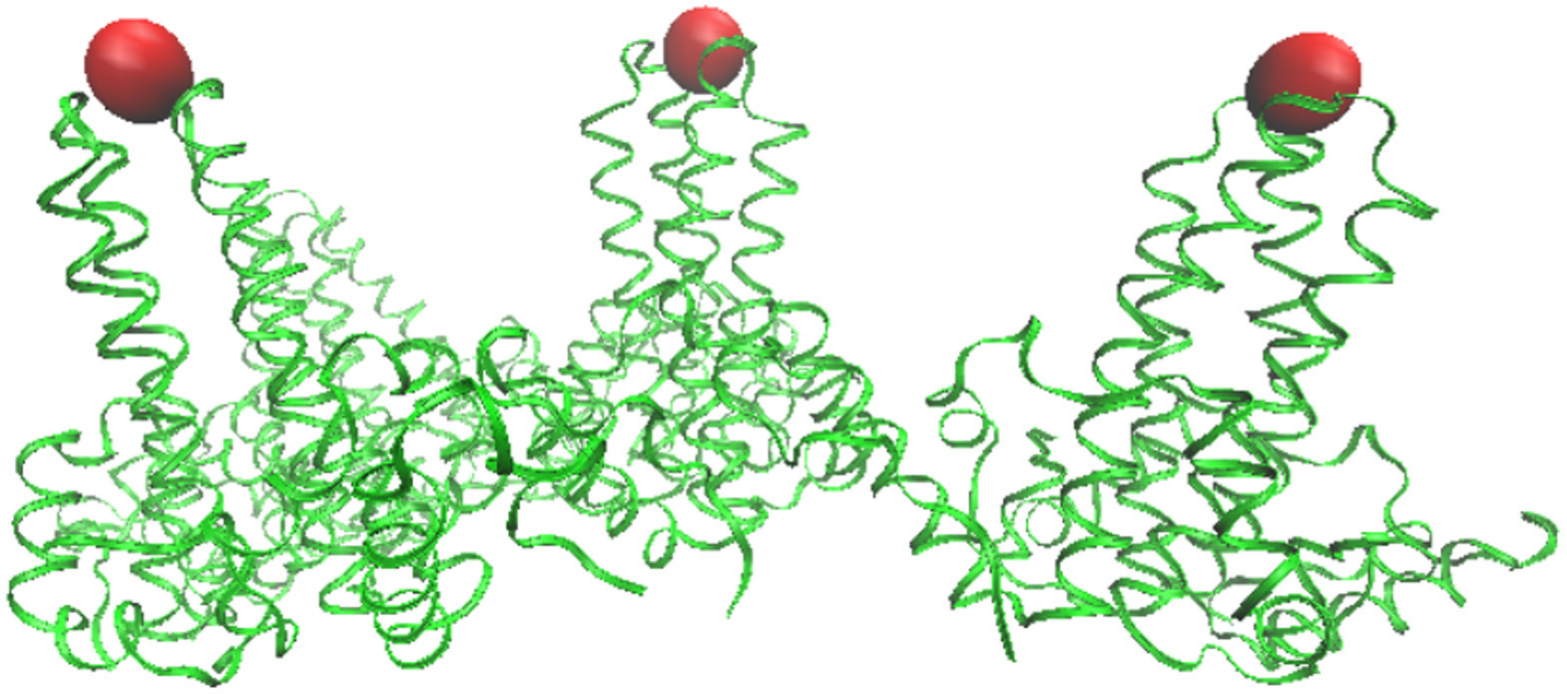
HepB Virus -a T4 virus with many helical bundles, which are perfectly aligned with the gauge points shown as red spheres. Both strains of HepB share the same protrusions which are the immuno-dominant regions. These protrusions are in excellent agreement with the gauge points. As HepB is eventually enveloped in its lifecycle, the exact spread of the helical bundles are likely important to its lifecycle.

Many viruses with distinct protrusion on the 5-3 plane also have a protrusion near the 2-fold, e.g. LA, TBSV, MS2 and GA virus. It also appears that T1 to T4 viruses seldomly have protrusions on the 5-fold. However some of these viruses, such as the homologous viruses MS2 and GA also have loops near gauge points 2 and 4 both on the 5-3 plane near the 5-fold. The most noteworthy and clearly distinguishable protruding features that are in excellent agreement with the gauge points are PAV, GA, MS2 and HepB, see Figs 8, 9 and 10. We have also found that all multi-protein protrusions, *e.g.* PAV, HepB, TBSV, have excellent agreement with the gauge points.

While we have just begun examining the location of spherical protrusions, we have yet to find any viruses with protrusions on the 3-fold axes, including the T1 capsids, which are dodecahedrons and one might expect them to have protrusions here. All known T1 capsid structures can be seen in the T-number Index on the Viperdb website, [3, 14], and none have 3-fold protrusions, although some do have protrusions near gauge point 5 and 7. There also appears to be a lack of protruding features along the 2-3 plane, with only one prominent exception, which is TBSV with a 3 protein coordinated tower at the 2-fold. The other two viruses with features on this plane are CCMV and MS2, however each of these viruses also has features along the 5-3 and 5-2 planes, see Fig 3. Quasiequivalence says that all hexamer proteins should be in nearly equivalent chemical environments across the capsid, the presence of protrusions along the 2-5 plane and lack of protrusions along the 2-3 plane, suggests a loss of quasi-equivalence in terms of protrusion placement, see Fig 1 for more details.

The proximity of protrusions to the gauge points appears to hold as T-numbers increase. This result is a remarkable result given that the packing density of proteins with respect to angular extent increases with T-number, while the angle between the pentamers remains fixed. Stated another way, there are ever more hexamers being packed into the same angular spacing, which should offer ever more diversity in the locations of protruding features, however this is not the case. As T-number increases, diversity in the location of protrusions decreases, leaving the majority of T7 capsids with protrusions only on their pentamers near the 5-fold axes. This result is expected to become inescapable for very large T-number viruses, as large spherical viruses seem to always have convex pentamers, requiring that the packing of additional hexamers be recessed from the maximum surface radius, and examination of the T-number Index on the Viperdb website, [3, 14] confirms this finding.

We will now by discuss the protrusions of T1 to T7 viruses and the patterns that we see within a particular T-number and across the range of T-numbers. We will then discuss the features in order of those found along the great circle connecting the 5-fold to 3-fold (Gauge points 1-6), then the great circle connecting the 3-fold to 2-fold axes (Gauge points 7-14) and finally along the 2-fold to 5-fold axis (GP 15-21). The location of each virus’s protrusions can be found in Table 2.

### T1 Capsids

The T1 viruses we examined were Adenovirus, PCV, STMV and L-A virus, a quasi T2. None of these viruses have protrusions along the 5-fold axes, however several do along the 5-3 plane, namely L-A and STMV at GP 3 and adeno-associated virus at GP 4. T1 viruses tend to have features along the 5-3 plane and the 2-5 plane. A few families of T1 capsids have features on or near the 2, 3 or 5 fold symmetry axes, such as Birnaviridae, Bromoviridae and Hepeveridae as can be seen in the Family Index on Viperdb [3], however none of the capsid we examined do. A few T1 capsids have features on the 2-3 plane near the 3-fold, but in general this region is unused. Two of the T1 viruses have two protrusions, one each along the 5-3 plane and the 5-2 plane. PCV and STMV each only have one protrusion, along the 5-2 plane and 5-3 plane, respectively. LA virus has two proteins arranged as a dimer within its AU, making it effectively a T1 capsid. It has two protrusions near GP 3 and 17.

### T3 Capsids

We have found that in general, T3 capsids have their protruding features along the 5-3 plane and near the 2-fold axis along the 5-2 plane. Additionally CCMV and MS2 also have features along the 2-3 plane, a hexamer protrusion for CCMV and a loop for MS2. In fact, only T-3 viruses have been found to have protruding features along the 2-3 plane. We analyzed CCMV native and swollen form, MS2, GA, PAV, TBSV and the pseudo T3 Seneca Valley. All of the T3 capsids have excellent agreement with the gauge points, the largest deviation being a hexamer protrusion in the model fit CCMV swollen form.

GA and MS2 are interesting viruses to consider as they have homologous protein and capsid structures. Each has 3 surface loops within the AU in nearly identical locations, yet the loop near gauge point 14 for GA is slightly bent over, making it closer to the surface of the capsid. This result serves as an example of why using the affine extended point arrays as a set of measuring tools is so powerful, as this difference between the loops of MS2 [15] and GA is easy to overlook using other tools, such as RMSD differences or molecular visualization tools, yet this detail is immediately apparent when using the gauge points to measure their locations.

### T4 Capsids

**Fig 11:**
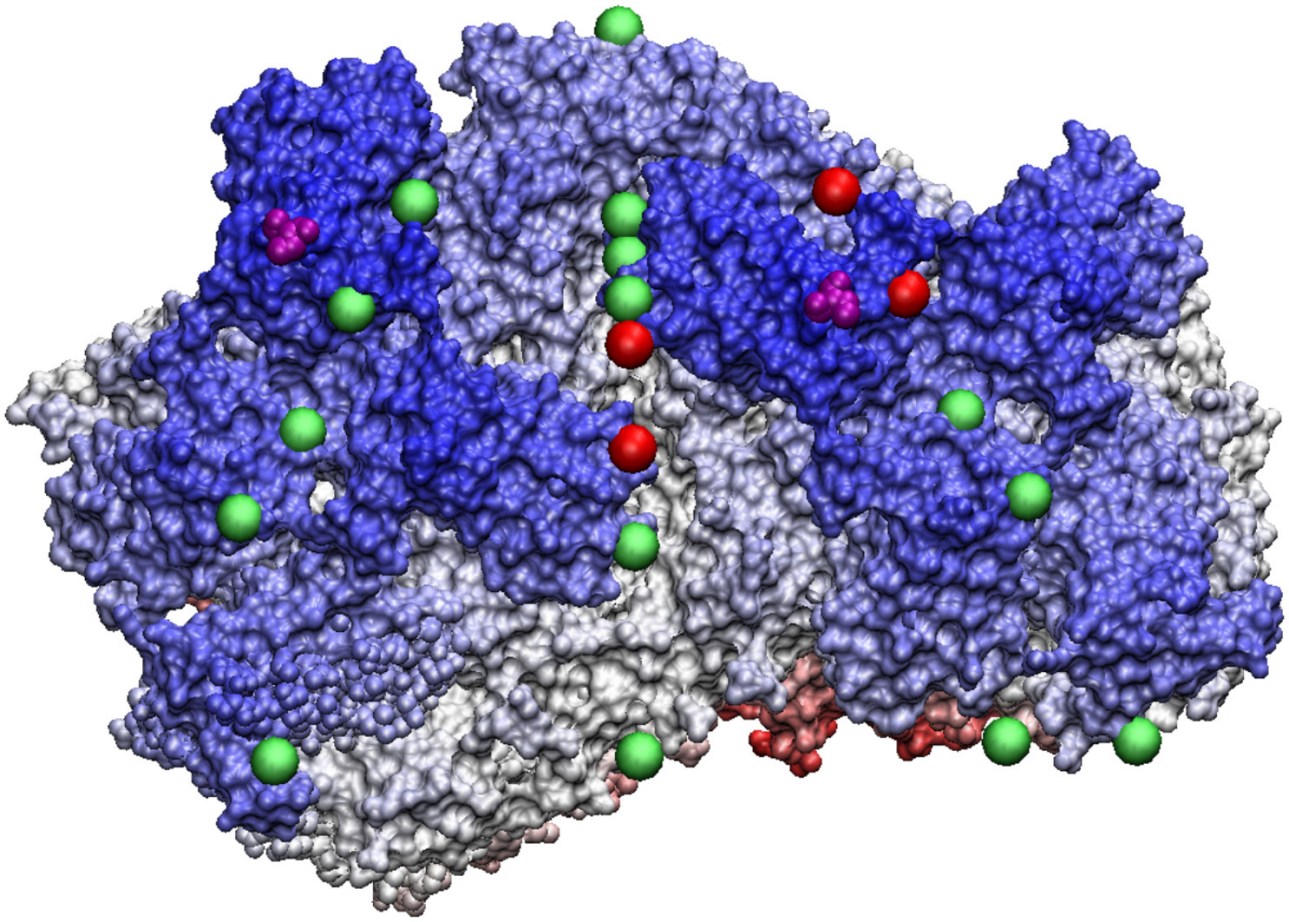
N*ω*V—is a T4 virus with protrusions (purple) that appears to have poor agreement with the gauge point constraints. The center of mass of its protrusions are far from the gauge points, see Table 2, however the dark blue regions are raised above the average thickness of the capsid and stretch between several gauge points (red spheres), perhaps attempting to conform to the constraints.

T4 capsids have the best and worst agreement of the spherical capsids. The best agreement with gauge points being the two strains of HepB, which each have multi-protein helical bundles situated precisely on the gauge points, with an angular separation of about 0.5°, see Fig 10. The worst agreement comes from two two related T4 capsids, N*ω*V and HASV which have very similar protruding morphologies, which leads to a large angular deviation of 5.9° and 6.2° degrees, respectively. The relatively large protruding bulges do not appear to respect the gauge points and each has a loop as its most radially distal feature. Close examination shows that these bulges however span the large solid angle stretching between gauge points 3 and 17, and appear bounded by them, see Fig 11. It is possible that their bulge protrusion are stretched between these two gauge points in order to adhere to an underlying fitness mechanism conferred by having protrusions at the gauge points.

### T7 Capsids

**Fig 12:**
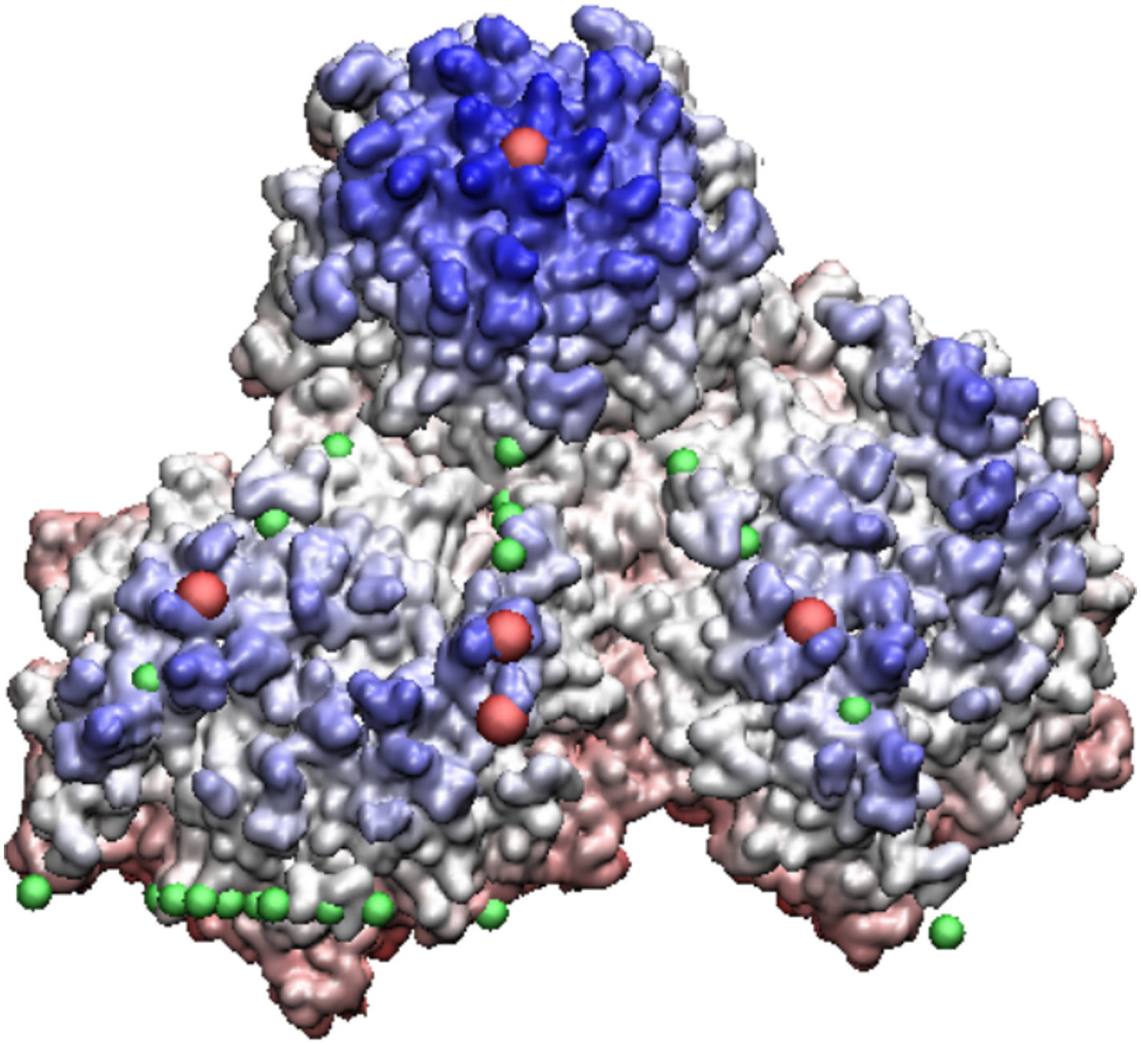
HK97 Prohead II—this relatively large T7 virus under goes a maturation to the Head II state, see Fig 13. Shown here are two of its hexamers (lower half of image) with their protrusions shaded in blue. The most radially distal protrusions are all located near the gauge points, shown in red. Quasiequivalence says the proteins should be nearly identical, however their protrusions are clearly not, as some are recessed into the capsid, seen as lighter blue. This result supports our suggestion of new restrictions for capsid modifications of protrusions.

**Fig 13:**
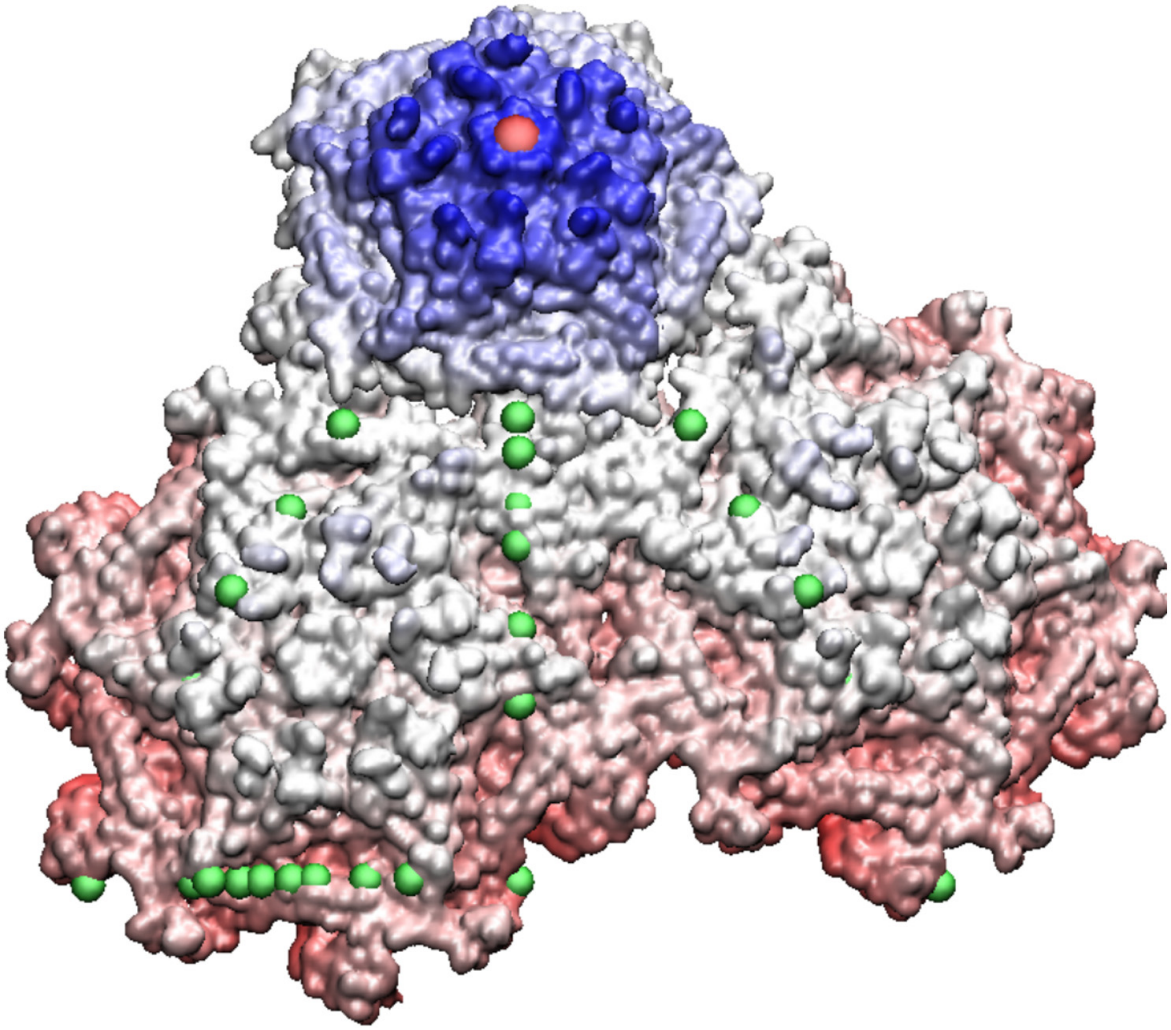
HK97 Head II—the mature form of HK97 Prohead II. When the capsid matures, it buckles and the hexamers recede into the capsid leaving the only external feature remaining on its pentamers. This is an example of maturation respecting the constraints of the gauge points.

All of the T7 viruses that we examined (HK97, BPV, SV40) show at least one protrusions along the 5-fold axes (GP 1), near the center of their respective pentamers. This result would be expected if the viruses were solid icosahedra, which envelops many larger spherical viruses. The immature Prohead II structure of HK97, see Fig 12 also has protrusions coming from its hexamers whereas the mature Head II structure of HK97 does not, see Fig 13. These protrusions are in excellent agreement with the gauge points. This result is of interest due to there being a lack of protruding feature quasi-equivalence in the hexamer, as the six proteins of the hexamer protrude in such away that the most radially distal portions line up with the gauge points.

**Fig 14:**
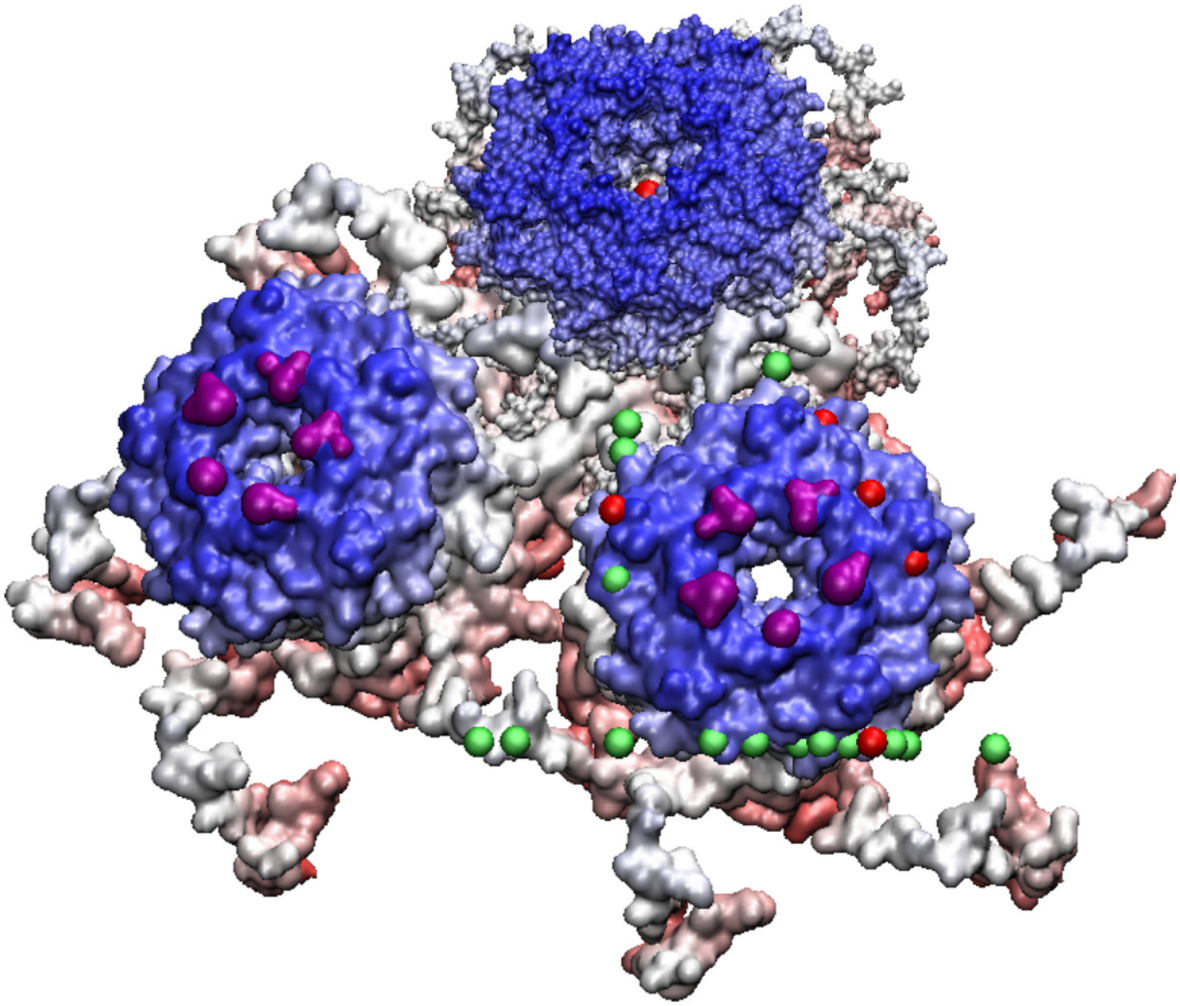
Simian Virus 40—is an unusual T7 virus that is entirely covered by pentamers instead of the usual hexamers and pentamers. Shown here are 3 of its pentamers, the upper pentamer is placed in the usual space along the 5-fold axes and is in agreement with the gauge point (red) while the remaining two pentamers are located where you would expect to find hexamers. The protruding features are shown in purple, and while collective their centers of mass are located in the center of the kite sections of the AU region, each protein considered individually (purple) would reduce the angular difference from the gauge points, and are perhaps each in agreement with the gauge points.

SV40 and BPV are T7d capsids of unusual construction. They are composed entirely of pentamers, instead of a mixture of 12 pentamers and 60 hexamers as would be predicted by their T-number. Also of interest is that these additional pentamers are not situated on 5-fold axis, and have pentameric protrusions which have a center of mass sitting in the middle of the triangular sections of the asymmetric unit, seemingly in violation of their constraints. However when we look more carefully, we see that each protein of the pentamer protrudes nearby a gauge point, see Figs 3 and 14. The pentamers coinciding with the 5-fold axes are a bit more spread out in angle than the other pentamers of the capsid, resulting in what appears to our algorithm as five separate protrusions; each appearing very close to the 5-fold axes, if these are treated as one large protrusion, the agreement is perfectly inline with gauge point 1.

### Virus Maturation

The location of protruding features also appear to conform with the gauge points through pH induced swelling maturation, as seen in CCMV and HK97. When the pH environment around CCMV is raised from 5.0 to 7.5 a [16, 17], it swells by approximately 10% in radius. While the swelling is not a uniform expansion, instead relying on modes along the 3-fold axes [17, 18], nevertheless the angular locations of its protruding features remain relatively fixed; with two features moving slightly closer to their gauge points and one feature moving slightly away, see Table 2. Conversely the maturation of HK97 occurs when we decrease the pH from 7.0 to 5.0. In this case, the Prohead II capsid swells and buckles to form the Head II structure [19, 20], see Figs 12 and 13. The hexamer protrusions of the Prohead II state are due to four proteins, the larger protrusions are near both gauge points 16 & 17 and the smaller near gauge point 5. After maturation, the hexamer protrusions recede relative to the 5-fold protrusion, see Fig 13.

## Discussion

It is not yet known why the protruding features of spherical viral capsids are found on or near the icosahedral great circles. The location of the protrusions strongly suggests that there is an energetic benefit or biological fitness, or mixture thereof conferred by adhering to these constraints, as there are many ways to build icosahedrally symmetry capsids without adhering to the gauge points constaints.

It is often stated that icosahedral symmetry is the largest discrete symmetry on a sphere, while this statement is true, it is also incomplete. The full icosahedral group includes mirror reflections, which due to chirality, is not applicable to viruses. It is worth pointing out however that these two groups have the same icosahedral great circles, see Fig 2, and while chirality prevents mirror symmetry, the locations of the protruding features being found only on the great circles may be the viruses attempting to adopt the full icosahedral symmetry of a discretized sphere.

The distribution of protrusions around the gauge points could be the result of maximizing the angle between features for the best coverage of the spherical capsid with the minimal number of features used, however this placement is not required by icosahedral symmetry. The symmetry dictates that you have sixty copies of any feature found within the asymmetric unit unless the feature is on a symmetry axis; Protrusions at these locations appear 12, 20 or 30 times for the 5, 3 or 2 fold symmetry axes, respectively. If a protrusion appears off the symmetry axes but on a great circle, *e.g.* the 3 protein spire of PAV, it will appear 60 times across the capsid. Whereas if the spire were located in the middle of the AU triangle section, it would still appear 60 times, as there is no mirror symmetry within the AU, see Fig 2. In fact the location of the features do not lead to a reduction of occurrences in general, *e.g* MS2 a T3 virus has 3 protruding loops within the AU, and therefore has 180 loops covering the capsid. To reiterate, the placement of protruding features along great circles is not required by icosahedral symmetry, and unless they are found on symmetry axes do not result in a reduction of surface features. It is more likely that their placement is the result of viruses conforming to affine extended icosahedral symmetry [10] and/or mirror symmetry.

Irrespective of why protruding features are arranged this way, the result suggests that when attempting to modify surface features on capsids for bioengineering applications, that one should consider where on the surface the modification will take place with respect to the gauge points, especially for modifications of the hexamers see Fig 1. As the number of hexamers increases, their relative orientations with respect to the pentamers shift, which in principle should allow the protruding features to be found nearly anywhere on the sphere, however we see that instead they remain on the icosahedral great circles. This suggests there are additional restrictions on how hexamers tile themselves on the sphere and perhaps new spatial restrictions in addition to normal the biochemical considerations. We suggest that it should be easier to modify features away from the 3-fold axes and closer to the 5 and 2-folds. When modifying hexamers, they should not be considered quasi-equivalent, instead the portions of the hexamer which are on the 5-3 great circle and 5-2 great circle should be targeted for modification. It has also not escaped the authors notice that there are likely evolutionary implications due to these restrictions, as it is likely that viruses evolved in a manner that drove them to conform with the structures permissible with these gauge points.

## Conclusions

We have shown that protruding features of spherical viruses will typically be found only on or near icosahedral great circles. This result was demonstrated using a set of gauge points derived from considering all allowable affine extensions of the icosahedral point groups [10]. By measuring the angular separation of the center off mass of each protrusion from the set of gauge points, we have shown that the gauge points serve as a natural toolset for characterizing spherical viruses.

The proximity of protruding features to great circles is significant as it is not required by icosahedral symmetry nor quasi-equivalence. There are many possible icosahedral capsid configurations which do not utilize these gauge point constraints and the majority of the 4*π* solid angle of the spherical area is not utilized for placing protruding features. This suggests that there is some underlying fitness criteria driving viruses to these configurations.

Many viruses (*e.g.,* HepB, L-A, MS2) show excellent agreement with the gauge points and their protrusions. These protrusions take several forms, *e.g.,* as loops, helicies, spires and peaks of hexamers. When more than one protein is involved with formation of the protrusion, the agreement is nearly perfect, *e.g.,* HepB, TBSV and PAV, with about 0.5° deviation from the gauge points. Even the few viruses with poor agreement *e.g.,* N*ω*V or SV40) have underlying structure to their protrusions which span multiple gauge points, indicating that they many be attempting to conform to the constraint as best as possible.

We find that smaller viruses (*T* < 7) tend to have their protrusions along the 5-2 and 5-3 planes, and rarely (if at all) on the 5-fold axis. We have also not found any viruses with 3-fold protrusions, including T1 capsids which are dodecahedral structures. As T-number increases, we expect that all protrusions will occur near the 5-folds due to geometric limitations of packaging more hexamers into the same solid angle and that viruses appear to always be convex on the 5-fold axes. We also find that pseudo-T number capsids conform to the gauge point constraints, even if they do it in an unusual way, such as SV 40 and BPV. We also see that virus maturation respects the gauge point constraints after the rearrangement of the protein subunits, as in the case of CCMV and HK97. All of these results seem to indicate an underlying stability or fitness criteria conferred by adhering to the constraints of the gauge points and thus the affine extended icosahedral structures.

Given many viruses have been previously shown to have affine extended icosahedral symmetry [10], which our gauge points are derived from, we suspect that most spherical viruses will also be found to also have this extended icosahedral symmetry which instills a radial component to the icosahedral rotational symmetry. This result would have profound implications on our understanding of virus construction and overall structure of virion, as this extended symmetry also applies to the genetic material contained within. We also believe that the location of protrusions will limit the available packing conformations of the entire virus, which will be explored in future works.

The biological function of protrusions conforming to the gauge points remains unclear. Given that a wide range of T-number viruses from many virus families conform to these constraints suggests there many an evolutionary constrain on the viruses as well. There are also implications for bioengineering of viral capsids as possible surface protein modifications should also take into account the geometric location of the modifications. Specifically the regions between the 5- and 2-folds as well as the 5- and 3-folds should be most successful, while the space between the 2- and 3-folds should be avoided as should any region not on the great circles nor near the 5-fold symmetry axes.
